# Review of potential medical treatments for middle ear cholesteatoma

**DOI:** 10.1186/s12964-022-00953-w

**Published:** 2022-09-19

**Authors:** Matthias Schürmann, Peter Goon, Holger Sudhoff

**Affiliations:** 1 Department of Otolaryngology, Head and Neck Surgery, Universität Bielefeld, Teutoburger Str. 50, 33604 Bielefeld, Germany; 2 Department of Medicine, National University of Singapore, and National University Health System, Singapore, Singapore

**Keywords:** Cholesteatoma, Chronic inflammation, Innate immune system, Adaptive immune response, Positive feedback loops, Precision medicine

## Abstract

Middle ear cholesteatoma (MEC), is a destructive, and locally invasive lesion in the middle ear driven by inflammation with an annual incidence of 10 per 100,000. Surgical extraction/excision remains the only treatment strategy available and recurrence is high (up to 40%), therefore developing the first pharmaceutical treatments for MEC is desperately required. This review was targeted at connecting the dysregulated inflammatory network of MEC to pathogenesis and identification of pharmaceutical targets. We summarized the numerous basic research endeavors undertaken over the last 30+ years to identify the key targets in the dysregulated inflammatory pathways and judged the level of evidence for a given target if it was generated by in vitro, in vivo or clinical experiments. MEC pathogenesis was found to be connected to cytokines characteristic for Th1, Th17 and M1 cells. In addition, we found that the inflammation created damage associated molecular patterns (DAMPs), which further promoted inflammation. Similar positive feedback loops have already been described for other Th1/Th17 driven inflammatory diseases (arthritis, Crohn’s disease or multiple sclerosis). A wide-ranging search for molecular targeted therapies (MTT) led to the discovery of over a hundred clinically approved drugs already applied in precision medicine. Based on exclusion criteria designed to enable fast translation as well as efficacy, we condensed the numerous MTTs down to 13 top drugs. The review should serve as groundwork for the primary goal, which is to provide potential pharmaceutical therapies to MEC patients for the first time in history.

**Video Abstract**

**Supplementary Information:**

The online version contains supplementary material available at 10.1186/s12964-022-00953-w.

## Plain English summary

Middle ear cholesteatoma (MEC) is a destructive and locally invasive ulcerated lesion in the middle ear driven by inflammation which occurs in 10 out of 100,000 people annually. Surgical extraction/excision is the only treatment strategy available and recurrence is high (up to 40% after ten years), therefore developing the first pharmaceutical treatments for MEC is desperately required. This review is focused on the connections between inflammation and MEC pathogenesis. These connections can be used as attack points for pharmaceuticals. For this we summarized the results of research undertaken over the last 30 + years. MEC pathogenesis can be described by specific inflammatory dysregulation already known from arthritis, Crohn’s disease or multiple sclerosis. A hallmark of this dysregulation are positive feedback loops of the inflammation further amplifying itself in a vicious circle-like manner. We have identified over one hundred drugs which are already used in clinic to treat other inflammatory diseases, and could potentially be repurposed to treat MEC. To improve and expedite clinical success rates, we applied certain criteria based on our literature searches and condensed these drugs down to the 13 top drugs. We hope the review will serve as groundwork for the primary goal, which is to provide potential pharmaceutical therapies to MEC patients for the first time in history.

**Supplementary Information:**

The online version contains supplementary material available at 10.1186/s12964-022-00953-w.

## Background

### Middle ear cholesteatoma: A disease without medical treatment

The middle ear cholesteatoma (MEC) is an inflammatory, destructive, and locally invasive middle ear lesion composed of proliferative keratinizing squamous epithelium and its subepithelial connective tissue. In general, there are two kinds of MECs. The rare congenital form arises from squamous epithelium trapped within the temporal bone during embryogenesis, and the more frequent acquired type of MEC which has an annual incidence between 7 and 15 out of 100,000 [1–4]. As disease progresses, the lesion becomes chronically and increasingly inflamed and this escalating inflammation leads to several complications. (1) Erosion of adjacent bony structures resulting in conductive and/or sensorineural hearing loss. MEC may lead to severe complications like (2) vestibular dysfunction, (3) facial palsy, (4) brain abscesses and other intracranial complications. Even though this severe disease was first described by Duverney in 1683, there is still no medical treatment, hence surgical removal of the MEC offers the only therapeutic possibility today [5]. Unfortunately, recurrence often occurs within 10 years in 40% [1] with even higher rates in children [6]. Rates of recurrent MECs are partly dependent on surgical techniques [7] and impacted by different approaches and techniques [8].

The main characteristic of MEC tissue is its abundant and chronic inflammation. It has been shown that the bone erosion as well as progression and recurrence are associated with the high degree of inflammation, which is also frequently linked to infection of the MEC tissue [9].

## Applicable targets in precision medicine for MEC therapy

### Methodology

Since chronic inflammation is the major source of MEC pathogenesis, this review will focus on the inflammatory intra- and extracellular signaling responsible for the etiology and pathogenicity of MEC. For this we use peer reviewed original research articles written in English as well as published review articles from the PubMed/Medline database between January 1990 and April 2022.

In the first section, we will focus on the signs triggering the primary inflammation as well as the intracellular signalling and the inflammatory output of the immune system residing in the MEC niche. In the second section we discuss the mechanisms by which inflammation drives osteolysis, epidermal proliferation and angiogenesis. All of this is aimed at the identification of molecular targets which can be applied in precision medicine. For this we only sum up studies which were able to draw connections between these targets and MEC pathogenesis. Wewill distinguish between in vitro, animal models and clinical data to judge the relevance of a specific cue/factor to the clinical outcome. In vitro evidence were derived from primary cultures mostly derived from MEC tissue, in vivo correlations were exclusively obtained from well-established animal models of MEC. Clinical data was derived from either cohort studies, used to detect the correlations between a certain target and the clinical outcome, or controlled clinical trials, always used to prove deregulation of this target in different control tissue compared to MEC. During this review we will focus solely on these fractions of the signaling pathways which are deregulated in MEC tissue and hence more accessible to pharmaceutical treatment compared to the surrounding healthy tissue. To facilitate the main goal of this review which is the identification of possible pharmaceutical treatment options, we consider only targets for which previous investigations showed a non-contradictory connection to MEC pathogenesis and those without available drugs within our inclusion criteria (Additional file 1: Table S1), described in the subsequent paragraph. Based on these descriptions, we will discuss possible medical treatments, aimed at the described roots and try to judge their potential to restore the proper function of the wrongly trained inflammatory network and their clinical potential. Indeed, based on the hierarchy of evidence described above, we derive a table marking the different drugs in accordance to the level of evidence. In this table we will suggest drugs, which have already been used for and applied to the middle ear and might be directly tested in clinical studies. Most importantly we proposed numerous drugs in the context of a molecular targeted therapy (MTT), which are already applied systemically in clinic but might previously be tested for their pharmaceutical effect on MEC in an animal model. For some targets numerous drugs are available. In this case we have suggested the ones which are already approved for clinical application. To enable fast translation into clinical use, the vast landscape of experimental modulators of inflammatory signalling only applied through an in vivo or in vitro model so far were ruled out. Also excluded were approaches based on RNA, lytic viruses or antibodies. Even though we think their topical application to an open wound-like tissue e.g. MECs might potentially work, this has never been tested and hence offers no fast track to translation.

By this we hope the comprehensiveness of this review inspires and streamlines new research into this topic, which might expedite the development of the first medical treatment option based on precision medicine, and effectively manages progression and recurrence of this hazardous lesion in numerous MEC patients.

### Targets from initial triggers of inflammation

The chronically escalated inflammation in MEC is known as a predictor for its pathogenesis and pathogenicity. Hence, in vivo studies have showed a decrease of MEC pathogenesis by systemic application of prednisolone used to treat various inflammatory conditions [10, 11]. Due to its ototoxic properties, these kinds of drugs are only applied topically and systemically in complicated cases with labyrinth fistula, brain abscess or meningitis [12]. But more recently the non-ototoxic drug montelukast usually used for maintenance treatment of asthma or allergic rhinitis [13], showed similar benefits. Therefore, a clinical study comparing local application of montelukast to traditional treatment, may enable new treatment options.

As already mentioned, this review focuses on a more targeted approach aimed at the characteristics of MEC disease. The main aspects of MEC tissue are tissue destruction, cell death as well as microbial infection. From these three sources the initial drivers of inflammation in MEC are derived. They can be grouped into the pathogen associated molecular pattern (PAMPs), which originate from bacteria, and damage associated molecular pattern (DAMPs), derived from stressed and dyeing cells (endogenous) and decomposing extracellular matrix (ECM; exogenous). Different studies have linked the PAMP Lipopolysaccharide (LPS), which originate from Gram (−) bacteria, to MEC formation [14] in patients, in vitro or in vivo MEC growth [15, 16], respectively and bone resorption in the clinic [14] as well as in an animal model [16]. But even though MEC often occur after chronic infection of the middle ear, clinical sampling has showed, that from nearly 40% of MEC, no bacteria could be cultivated [17]. But more recent studies comparing metagenomics approaches with conventional culture-based techniques, have demonstrated an improvement of the bacteria detection rate to 100%, hence MEC cannot be considered sterile [18, 19]. We assume, that even though MEC tissue is not sterile, the exact composition of the microbiome may define and contribute to the clinical outcome. For example, the concentration of PAMPs like lipopolysaccharide (LPS) in MECs ranged over 4 orders of magnitude [14]. Even though fungal infection of the middle ear is a very rare entity, which is almost exclusively seen in immunocompromised patients, the microbial genus most often detected in these studies has been found to be Aspergillus, and this fungus was most frequently found by culture based approaches [20, 21]. Interestingly, Aspergillus presence could also be linked to severe bone erosion in patients [18]. In any case, other mechanisms triggering the initial inflammation (particularly in the non-infected MECs) must also be present. Since tissue damage is characteristic for MEC disease, various DAMPs are upregulated and could be linked to osteolysis in an animal model [22, 23].

The prevention of the generation of endogenous or exogenous DAMPs is directly coupled to prevention of MEC. Hence, a drug inhibiting MEC formation will additionally cause a negative feedback loop further reducing DAMP driven inflammation. PAMPs on the other hand, are directly linked to the presence of pathogens and are responsible for the signalling through the macrophage inducible Ca2 + -dependent lectin receptor (Mincle), which is able to detect PAMPs derived from fungal or bacterial sources, or Toll-like receptor 4 (TLR4) responding to bacterial PAMPs both characteristic for MEC. For bacteria, empirical broad-spectrum antibiotics are commonly applied post-surgery to prevent postoperative infection [24–26]. Due to the rise in antibiotic resistant bacteria (“super-bugs”), recent studies have shown that the application of specific drugs according to antibiotic susceptibility testing improved the rate of dry ear after surgery significantly [27, 28], a predictive factor directly coupled to MEC recurrence and bone destruction [29, 30]. This suggests that a clinical study comparing broad-spectrum antibiotic treatment vs antibiotic susceptibility testing based treatment with longer follow-up periods will be able to identify parameters like recurrence, bone destruction etc. and may encourage surgeons to utilize antibiotic susceptibility testing before surgery. Since fungal infection and Mincle are coupled to MEC pathogenesis as well, application of antimycotics might yet be an additional experimental arm in this study.

Unfortunately, not only certain DAMPs and PAMPs are upregulated but their receptors are also expressed at a higher level compared to healthy tissue, further exaggerating the inflammatory signalling in MEC tissue. A major contributor in terms of DAMPs is the upregulated receptor for advanced glycation end product (RAGE) [31], for which S100A8/A9 and particularly HMGB1 can serve as ligand, both known to be upregulated in MEC [31–33]. In vitro studies could therefore potentially prove, that the adverse combination of the receptor and agonist upregulation of the HMGB1/RAGE pathway, contributes to MEC pathogenesis [31].

The DAMPs and PAMPs recognizing TLR4 have been found to be of particular importance in regards to MEC disease. Hence various investigations utilizing transcriptomic and proteomic techniques were undertaken and demonstrated an upregulation of TLR4 in comparison to various different control samples e.g. “normal” control middle ear samples without any inflammation [34], congenital MEC [35] or external auditory canal skin [36–38] and even normal tissue from chronically infected middle ear [37]. As already mentioned TLR4 does not only recognize DAMPs already upregulated e.g. HSP27 [39], HSP60 and HSP70 (both 40), HMGB1 [31] and S100A8/S100A9 [32, 33] but can also be activated by PAMPs derived from Aspergillus and Candida or LPS, which are particularly abundant in purulent MECs [14, 21]. Unfortunately, an investigation utilizing Förster resonance energy transfer (FRET) measurements could detect a further amplification of the TLR4/LPS signalling through HSP70 by trafficking TLR4/LPS to the Golgi apparatus [41]. Various in vitro assays have suggested that TLR4 signalling is involved in the establishment of the inflammatory environment of the MEC niche [42] and MEC recurrence and proliferation [43]. Clinical data, as well as a Knock-out mouse model of acquired MEC directly linked TLR4 to the local inflammation and bone destruction [35]. TLR4 signalling is further enhanced by the triggering receptor expressed on myeloid cells-1 (TREM-1), which is upregulated compared to healthy control skin [44]. TREM-1 senses DAMPs like HMGB-1 and further amplifies the already exaggerated TLR4 signalling in vitro [45]. A second way to enhance the TLR4 signalling in MEC, might be the presence of macrophage migration inhibitory factor (MIF). Since MIF is able to induce TLR4 expression [46], the recurrent and osteolytic features of MEC disease may also be linked to the expression of MIF [47]. Due to the parallel activation of TLR4 and RAGE signalling in MEC and the interconnection between RAGE and TLR4 signalling on the intracellular level, these signalling inputs are able to bilaterally enhance each other. In this context, antibody blocking assays and animal models have shown that HMGB1 induced RAGE signalling increases the inflammatory potential of LPS in macrophages, one of the main immunomodulatory cells in MEC tissue [48]. A study which investigated various other PAMP recognizing receptors at the mRNA level found that Mincle, which is able to recognize PAMPs of bacterial or fungal origin respectively, is upregulated in comparison to external auditory canal skin and that its expression could be correlated to recurrence and osteolysis in MEC disease [49]. There are many more PRR receptors which are expressed in MEC tissue. But in contrast to TLR4, TREM-1, RAGE and Mincle, their contribution to MEC pathogenesis has not been proven so far.

**Fig. 1:**
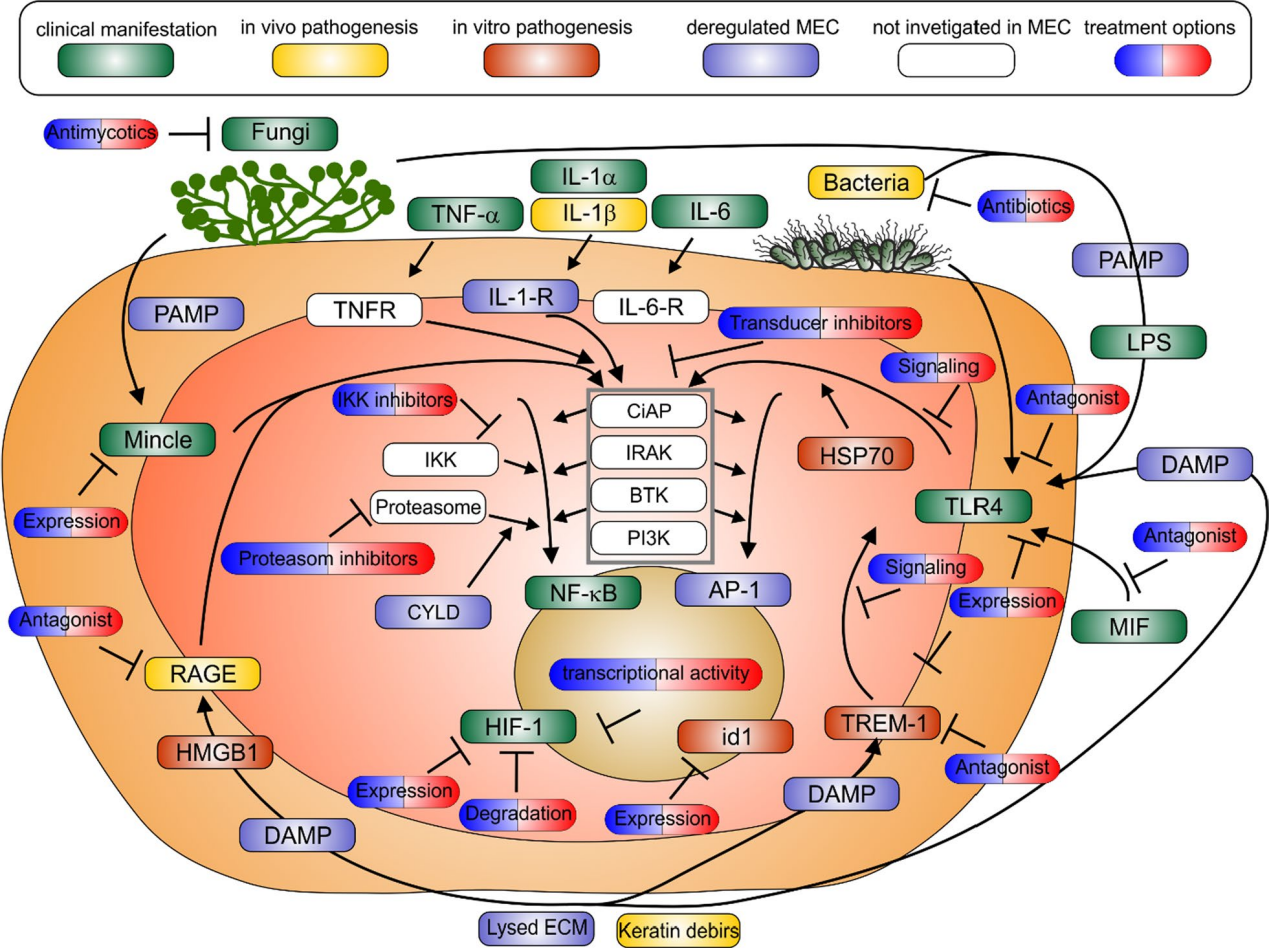
The proinflammatory intra and extracellular signalling in MEC disease. The orange background symbolizes the MEC, red represents the cytoplasm of residing cells and brown their nucleus. The level of evidence linking the depicted part of the pathway to MEC parthenogenesis are color-coded (red, yellow and green), proinflammatory deregulation is shown in blue. Fractions which were not investigated so far but bound to be a vital part of the proinflammatory signalling in MEC are shown in white. The pharmaceutical treatment options accessible within the inclusion criteria chosen in this review are shown as blue/red pills

Since it is hard to prevent PAMPs and particularly DAMPs in MEC, an inhibition of activation of PRR should be one of the first targeted approaches (Fig. 1). As described above TLR4 signalling is paramount in MEC pathogenesis. Interestingly, amitriptyline, a drug usually administered against migraine, inhibits the expression of TLR4- receptor and subsequent NF-kB signalling [50]. The up regulation of TLR4 expression, through MIF, might be dampened by Ibudilast known to inhibit MIF [51] and already applied against progressive multiple sclerosis [52]. Also, the MIF receptor antagonists RTL1000 [53] was evaluated for treatment of MS [54, 55]. After downregulating TLR4 expression the prevention of the activated (TLR4-MD-2-PAMP/DAMP)_2_ complex is the next pharmaceutical target. In vitro studies from our group, showed that LPS based MD-2-TLR4 antagonists might be one pharmaceutical approach against MEC [42, 43]. The Antagonists eritoran can be used to treat septic shock [56, 57], JKB-122 against hepatitis [58], VB-201 for the therapy of psoriasis [59], ibudilast [60] or dalcetrapib used for cholesterol management [61] and curcumin, for which topical application was shown to be effective against some inflammatory skin disorders [62]. An MD-2-TLR4 antagonist established in clinical practice are Taxanes [63] known from oncologic therapies [64]. But even though the (TLR4-MD-2-PAMP/DAMP)_2_ might be formed, targeted inhibition of downstream (MyD88-dependent and MyD88-independent) pathways offers an additional anti-inflammatory strategy. Resatorvid is a selective inhibitor of TLR4 which inhibits autophagy and is used for various inflammatory situations, which include neuroprotection following brain injury, the treatment of severe sepsis [65] or alcohol-related cirrhosis, and also inhibits both MyD88-dependent and MyD88-independent pathways [66]. The compound resveratrol used against arthritis or COPD [67] and alogliptin against Typ-2-Diabetes [68] blocked MyD88-dependent signalling [69, 70], respectively. Naltrexone and naloxone, drugs well known as opioid antagonists but also applied against inflammatory disorders like Crohn’s disease and neuroinflammation respectively, are MyD88 independent inhibitors of TLR4 signalling [71]. To inhibit the amplification of TLR4 signalling through TREM-1, its transcription can be inhibited by curcumin [72], or the blocking of TREM-1 ligand binding domain can be achieved by peptides like nangibotide already applied against septic shock [73–75]. RAGE can be targeted by extracellular inhibitors e.g. azeliragon applied against Alzheimer disease [76]. To target Mincle, quercetin, effective against upper respiratory tract infections [77], or curcumin [78], were shown to inhibit Mincle induced macrophage inflammation [79].

### Targets from extra- and intracellular inflammatory signalling pathways

As a consequence of the upregulated PRR-signalling, a proinflammatory environment is created with IL-1α, IL-6 and TNF-α being highly upregulated in the inflamed MEC tissue [80, 81]. Apart from the elevated expression of IL-1β, the corresponding Interleukin-1 receptor (IL-1-R) is upregulated threefold in MEC epidermis compared to the normal aural skin [82]. This pro-inflammatory signalling induces several intracellular signalling events characteristic for MEC tissue. The main transcription factors involved in this are NF-κB, AP-1 and HIF-1. Investigations have showed, that the expression of NF-κB was upregulated and more importantly the nuclear translocation, as well as the DNA binding activity were increased in epithelium of MEC tissue compared to healthy skin [83]. Furthermore, a significant correlation was found between TLR4 and activated NF-κB [37] in MEC tissue, and we assume, that the same could be shown for RAGE as well. Further, another study demonstrated a correlation between NF-kB and biomarkers of angiogenesis and osteolysis in MEC tissue isolated from patients [84]. In addition to this, the DNA-binding protein inhibitor Id1, known to be induced by chronic TLR4 signalling [85], was proven to be abundantly expressed in MEC epithelium [86] where it accelerates proliferation and survival of keratinocytes in vitro via NF-κB signalling [87]. Two studies found that the transcription factor AP-1 was detectable in the nucleus of MEC epithelium [88], where it was much more abundant compared to healthy ear canal skin [89].

Since hypoxia is common in MEC tissue, the expression of hypoxia-inducible factor 1 (HIF-1) was significantly increased in MEC compared to normal skin [90] and localized to keratinocytes located on the basal layer [91]. Interestingly, the likelihood of relapse after surgery was correlated to HIF-1 expression [90]. Further, downstream of the PRR and cytokine receptors, the transcription factor NF-κB is the most promising contributor to angiogenesis and osteoclast activation in MEC. Since NF-κB is a common target of various diseases, numerous inhibitors have been designed and clinically tested (reviewed in 92). Even though the role of AP-1 has not been not elucidated so far, AP-1 shares many upstream inducers with NF-κB. Hence both transcriptional factors can be inhibited by the same signalling transducing protein inhibitors (Fig. 1). Particularly interesting drugs for MEC are inhibitors of Bruton’s Tyrosine Kinase (BTK), since BTK lies downstream of TLR4, TREM-1, IL-1-R and TNFR, the dominant links between extracellular and intracellular signalling in MEC. The BTK inhibitors acalabrutinib [93], ibrutinib [94], zanubrutinib [95] and tirabrutinib [96] have been developed and applied to treat oncologic diseases but also various inflammatory skin diseases [97].

Another protein downstream of TLR4 and IL-1-R is Interleukin-1 receptor-associated kinase (IRAK). The inhibitor PF-06650833 developed against rheumatoid arthritis [98] is useful for inhibition of IRAK. Downstream of TLR4 and TNFR is the cellular inhibitor of apoptosis proteins (c-IAP), which can be targeted by inhibitors like birinapant usually used against solid tumors [99]. Pi3K Phosphoinositide 3-kinases PI3Ks, are a family of enzymes downstream of TLR4, TNFR and IL-6-R. Their inhibition by idelalisib [100], copanlisib [101] or duvelisib [102] approved for the treatment of lymphoma and alpelisib [103] useful against breast cancer. Directly upstream of the activation of NF-kB is IKK a/b, of which the inhibitors CHS-828 [104] have been used against solid tumours and VGX-1027 [105] for the treatment of rheumatoid arthritis. In the canonical pathway, proteasome degradation of IkB is crucial for the release of active NF-kB. Many useful proteasome inhibitors have been already applied in clinical practice e.g. disulfiram against alcohol dependence [106], ixazomib [107], carfilzomib [108] and oprozomib to treat multiple myeloma [109] or marizomib developed against glioblastoma [110].

HIF-1 can be inhibited by several drugs already in clinical practice, which act on different levels (reviewed in 111, 112). The accumulation of HIF-1 is effectively targeted by either enhancing its degradation with the drugs panobinostat [113], vorinostat [114], geldanamycin [115] and its derivative tanespimycin [116] applied against solid tumours and haematological malignancies or resveratrol used to treat against arthritis or COPD [117]. Another approach would be the inhibition of its expression with EZN-2208 [118] or EZN-2968 developed against solid tumours and lymphomas [119] or panzem which was additionally used in rheumatoid arthritis [120]. Interestingly panzem reduces HIF-1 transcriptional activity as well, hence it could attack HIF-1 signalling on two levels. Despite promising results, clinical trials with panzem were dismissed, due to its poor oral bioavailability. But *topical* application to MEC might revive the clinical prospects for this promising drug. Alternatively, inhibition of HIF-1 transcriptional activity can be achieved by echinomycin [121] or amphotericin B [122], known for its antifungal effect additionally reducing Mincle signalling. Finally, the drug acriflavine [123] can be used to dampen HIF-1 signalling by preventing HIF-1a/HIF-1b dimerization and is also able to inhibit the growth of Gram (+) bacteria as well, reducing microbial driven inflammation on two levels.

Even though Id1 can only be linked by in vitro models to epidermal proliferation as well as angiogenesis of MEC, it might be inhibited by many clinical approved drugs (reviewed in 124). Chief among these were fucoidan [125] and berberine [126], both inhibiting the expression of id1 and showing good effect against inflammatory disorders.

In conclusion, it is clear that numerous PAMPs/DAMPs and cytokines are abundant in MEC tissue and can be linked to promotion of MEC characteristics via establishment of an inflammatory environment. Importantly, only signalling via LPS/TLR4/NF-κB was demonstrated to contribute to the initial establishment of the inflammatory state of MEC disease as well as to its clinical outcome. In accordance with our criteria judging the quality of a target, we suggest that this pathway might be the most promising pharmaceutical target.

### Specific targeting of Innate immunoregulatory events in MEC disease

Due to its highly inflamed sub-epithelial connective tissue, middle ear MEC is characterized by a large infiltration of inflammatory cells. Macrophages are the subset of myeloid-derived blood cells defined here precisely as CD3+ and CD68+ cells, which have been found to be upregulated by a factor of about five in MEC showing high level of bone destruction compared to external auditory canal skin [127]. Other studies utilizing MEC tissue of all levels of invasiveness, have detected a two to fourfold increase of CD11c and CD68 positive cells in relation to normal ear skin and characterized them as dermal macrophages [127, 128]. We hypothesize that the polarization of MEC macrophages is likely to be of the proinflammatory M1 type, which is demonstrated by the high expression of nitric oxide synthase [129] and proinflammatory cytokines [80] in MEC. Another myeloid cell type, dendritic cells, were also increased in acquired MEC compared to congenital MEC [130] and their presence could be linked to drivers of osteolysis in MEC tissue in an animal model [131].

After infiltration, the differentiation of monocytes into proinflammatory M1 macrophages and conventional dendritic cells should normally be inhibited. There are several drugs already applied in clinic able to inhibit this polarization of macrophages in inflammatory disorders e.g. curcumin [132], bilobalide used against complications in pregnancy [133], quercetin already applied against COPD [134, 135], berberine [136] or rosiglitazone [137] known for their positive effect on diabetes, arctigenin [138] which is suspected to lower the risk of exaggerated inflammation. In regards to the dendritic cells abundant in MEC, substances like corticosteroids, long known for their application against inflammatory disorders [139], rapamycin, an immune modulator useful in helping prevent transplant rejection [140] or cyclosporine [141], known as a potent T-cell inhibitor applied in transplant situations where high immunosuppression is required, but also used against psoriasis, are capable of inducing the differentiation into tolerogenic dendritic cells priming the immune system into tolerogenic state against various antigens. Another myeloid cell type, the mast cells, are known to influence multiple features of persistent inflammation (reviewed in 142). In accordance with that, investigations showed a three-to sevenfold increase in MEC in relation to normal epidermal tissues [143], which are localized close to the epithelium [144] and to the eroded surfaces of the bone [145]. Since mast cells are known to influence various aspects of bone absorption, and particularly osteoclastogenesis, by histamine (reviewed in 146), we suggest they also increase osteolysis around MEC. In addition the released histamine is known to favour a proinflammatory response [147] further enhancing this phenotype in MEC tissue.

Due to its impact on the symptoms of inflammatory diseases, various drugs have been developed to inhibit the degranulation of mast cells e.g. in allergic conjunctivitis (reviewed in 148). The most popular are sodium cromoglycate, also applied against asthma and allergic rhinitis or the more potent lodoxamide, nedocromil as a prophylactic in asthma, ketotifen is also used against inflammatory conditions like allergic rhinitis and conjunctivitis, and chronic spontaneous urticarial. and the popular olopatadine additionally useful to treat allergic rhinitis. The subsequent inflammatory response can be inhibited by the vast numbers of clinically approved H1-antihistamines [149, 150] acting mainly on Th1 cells and M1 macrophages [151] with particularly the second generation having less side effect on the nervous system and should be preferred in middle ear application. Even though usually applied to treat hay fever, cetirizine, fexofenadine and loratadine were also effective against urticarial diseases and might be a treatment opportunity for MEC as well. Interestingly, ketotifen and olopatadine work as histamine 1 receptor antagonists as well and hence might attack histamine on two levels [148]. The clinically approved H2-antihistamines like nizatidine are known to reduce exaggerated inflammation after burn injury [152], and mainly reduce Th1 driven inflammation while newer H4-antihistamines (reviewed in 153, 154) mainly act on the mast cells to prevent their activation. Hence H4-antihistamines like toreforant have been used in asthma and psoriasis [155], JNJ39758979 used in clinical trials to treat symptoms of atopic dermatitis [156], ZPL-3893787 tested against atopic dermatitis and psoriasis [157] and UR-63325 developed to treat atopic dermatitis [156] might offer new opportunities.

### Pharmaceutical targets within the lymphoid cell regulation in MEC

As regards lymphoid lineage cells in the MEC, T lymphocytes are much more abundant in acquired MEC tissue compared to congenital MEC [158] or normal ear skin [127, 159]. Different studies aimed at delineating the Th1/Th2 paradigm of inflammatory events in MECs, found that according to the expression profile showing TNF-α, IL-1β and IL-6 and lacking IL4, IL4R, IL10 and IL10R, the Th_1_ cells were likely to be dominant [80, 144].

The differentiation of T_0_ cells into proinflammatory Th_1_ cells and their subsequent activation is another major proinflammatory cellular process in MEC. The differentiation can be blocked by clinically approved drugs like beta2-agonists, a group of drugs with a long history prescribed for asthma [160, 161] (with a wide range of different drugs) (reviewed in 162). Other drugs include progesterone applied against endometriosis [163], glucocorticoids known for their potency in suppressing various allergic, inflammatory, and autoimmune disorders [164], flavocoxid which has proven useful against osteoarthritis [165], epigallocatechin gallate used to treat multiple sclerosis [166] or phosphodiesterase-4 inhibitors regularly utilized in the case of inflammatory diseases like asthma, COPD, atopic dermatitis and psoriasis [167–169]. To reduce the activation of Th_1_ cells and inhibit Th_1_ cytokine secretion, the clinically approved drug Vitamin D3 [170] known as treatment primarily against osteoporosis (or any cause of Vit D deficiency) can be used.

The large amount of infiltrating cells in MEC tissue is likely due to the overexpression of the intracellular adhesion molecule-1 (ICAM-1) found in vessels of acquired MEC compared to normal skin [171], congenital MEC [158] and ear canal skin, tympanic membrane or facial skin (all [172]. Studies have linked the high expression of ICAM-1 to the level of inflammation in MEC [158] and in vitro studies have demonstrated that human dermal microvascular endothelial cells expressed ICAM upon stimulation with typical M_1_/Th_1_ cytokines e. g. IL-1α, IL-1β, and TNF-α [173] all of which are highly upregulated [81, 174]. We assume that this establishes a spiral of doom of self-enhancing inflammation, in which there is enhanced migration of Th_0_ and M_0_ cells into the tissue, their differentiation into M_1_/Th_1_ cells further increases the inflammation, and this upregulates ICAM-1 which again results in even more immune cell migration and inflammation respectively.

The enhanced expression of ICAM in MEC endothelium cells, responsible for increased infiltration of MEC tissue, might be inhibited by several clinically applied drugs like resveratrol [175] or another rheumatoid arthritis drug called methotrexate [176], curcumin [177] and especially alicaforsen which showed promising results against pouchitis and different kinds of inflamed colo-rectal conditions [178].

Since T-cells seem to play a key role in the progression of inflammation in MEC disease, the role of Human Leukocyte Antigen-DR isotype (HLA-DR) was also more closely investigated in the context of MEC disease. In general, HLA-DR functions as MHC class II cell surface receptor presenting e.g. bacterial antigens to T-cells. HLA-DR is upregulated [144, 159] and abundantly expressed in macrophages present in the perimatrix of MEC tissue [179, 180]. The high expression of HLA-DR on macrophages in combination with the abundance of e.g. bacterial antigens results in the activation of the frequently found T-cells and is thought to be an additional source of the immunologically activated state of the infiltrating cells observed in MEC tissue [180, 181]. To reduce HLA-DR expression, Iscador usually applied against solid tumors can used [182].

**Fig. 2:**
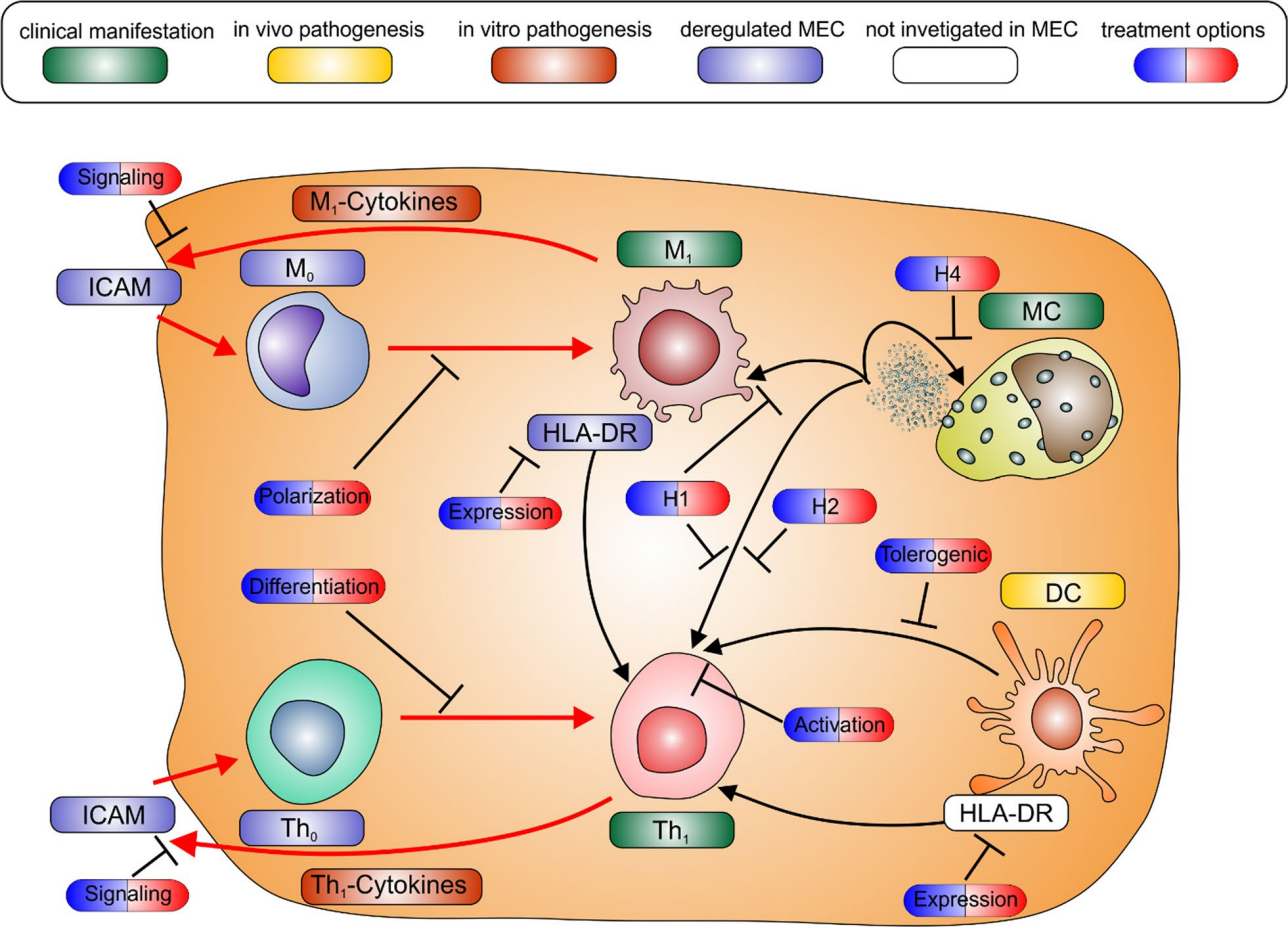
Regulation of Immune cell response in MEC. The colour coding is the same as in Fig. 1. Highlighted in red are the spirals of doom circles amplifying and exacerbating the already heightened inflammatory situation in MEC

It can be said definitively that the M_1_ and Th_1_ cells residing in MEC tissue are major contributors of inflammatory cytokines and antigen presentation via macrophages and dendritic cells to Th_1_ cells is the most frequent immune regulatory event in MEC tissue (Fig. 2). All this is crucial in maintaining the chronic inflammatory state of the MEC niche responsible for the clinical symptoms described in the upcoming chapters.

All the approaches mentioned above target the reduction of the proinflammatory MEC signalling initially triggered by PRR and amplified by proinflammatory extracellular signalling. In the second section we want to focus on the two major symptoms of MEC disease the osteolysis and the uncontrolled proliferation of keratinocytes and MEC mass supported by angiogenesis and their applicability as target in MEC therapy. We want to remind our readers, that this strategy not only targets the symptoms but more importantly, eliminates the “fuel to the fire” of the ongoing inflammation, the DAMPs derived from keratin debris and bone matrix.

### Directly targeting links between inflammation and drivers of osteolysis

As mentioned above conductive hearing loss is a frequent and serious complication caused by MEC disease. It can result from tympanic membrane rupture and/or changes in the ossicular chain due to fixation or most often bony erosion caused by the chronic inflammatory process [183]. Today there are two different mechanisms suspected to be relevant in the process of bone resorption by MEC, the (1) abundant osteoclast activation and (2) enzymatic digestion. These mechanisms are triggered and enhanced by the severe inflammation in a positive feedback manner as discussed. In the upcoming section we will highlight the main influences behind these mechanisms and their contributions to the clinical outcome.

A previous histo-pathological study detected an increase in osteoclast density [81] and other studies have directly linked osteoclast activation to bone destruction found in MEC disease [81, 184]. For osteoclast activation, the receptor activator for nuclear factor kappa B ligand (RANKL) has been identified as a key factor in promoting the differentiation and fusion of osteoclast precursor cells and activating the bone resorption by mature osteoclasts, while osteoprotegerin OPG on the other side is a decoy receptor for RANKL negatively regulating this process [185]. In accordance with this RANKL-positive cells and/or the ratio of RANKL/OPG were significantly higher in MEC compared to every other skin sample e.g. external meatal skin [186, 187], post auricular skin [188] normal skin [189], auditory canal skin and even granulation tissues [190] and RANKL expression positively correlated with osteolysis in MEC patients [186, 191]. In contrast to the upregulation of RANKL in the soft body tissue adjacent to the decomposing bone, there was no evidence found for an increase in RANKL or RANKL/OPG ration inside the bone tissue more distant to MEC [192]. Various investigations have tried to shed light on the source of RANKL in MEC. They found, that overexpression of RANKL is typically expressed by activated T-cells [193, 194] abundant in the perimatrix, but also from epithelial [186, 191] and fibroblastic cells [81, 186].

A paracrine positive regulator of RANKL signalling in MEC, the parathyroid-hormone-related protein (PTHrP), is usually expressed in keratinocytes of the skin. Since PTHrP is known as an inflammatory marker [195], keratinocytes from MEC express more PTHrP than normal keratinocytes [196]. Most recently a study demonstrated, that not only protein expression of PTHrP in MEC epithelium is significantly increased but also positively correlated with the expression of RANKL in osteoblasts [197] and correlated to the degree of bone resorption [191]. Another source of RANKL is known from rheumatoid arthritis, where the RANKL-Th17 system contributes to bone destruction in a paracrine manner. Another study showed that IL-17 immunoreactivity was increased and was localized to CD4+ lymphocytes. Most importantly this correlated with the number of cells positive for RANKL and the degree of bone destruction [198]. We assume that TNF-α and INF-γ secreted by the numerous Th_1_ cells triggers the production of IL-23 in macrophages, and IL-23 ultimately differentiates Th_0_ into Th_17_ cells which further enhances osteolysis in MEC disease, by priming fibroblasts and maybe osteoblasts with IL-17 and thereby inducing RANKL in a paracrine manner.

As described in the previous paragraphs, the main driver of osteolysis is RANKL derived from inflamed keratinocytes, fibroblasts and Th_17_ cells. Inhibition of RANKL signalling in clinical trials by small molecules was achieved by bisphosphonates developed against osteoporosis [199], AZD4547 a new anti-cancer drug [200], isoflavone (useful against asthma) [201], and iguratimod, used to treat rheumatoid arthritis [202]. The widely used bisphosphonates are able to suppress RANKL expression [203]. PTHrP derived from epithelium inducing RANKL expression in osteoblasts might offer an alternative target in the MEC context. The clinically approved antitumor agent cabozantinib decreased PTHrP expression and might be useful in this context [204]. The secretion of another factor responsible for osteoclast differentiation, IL-17, can be reduced by clinically applied drugs KD025 [205, 206], fused pyrimidines [207] or iguratimod [208].

In vitro studies on primary MEC epithelia cells demonstrated, that expression of RANKL can be induced by stimulation of the TLR4 by LPS [209]. However, since clinical data demonstrated no correlation between RANKL expression and bacterial infection of the MEC [17], the correlation between osteolysis and LPS, as well as TLR4 might have another source. Hence the connection between proinflammatory cytokines and osteolysis in MEC was further investigated in various studies. In vitro studies showed, that LPS initiated expression of TNF-α, IL-1β and IL-6 via TLR4 pushed the precursor cell in an autocrine manner, and primed with sub-osteoclastogenic doses of RANKL, into fully differentiating phases [210]. This is in accordance with the clinical finding, that IL-1α, IL-6 and TNF-α are highly upregulated in the inflamed MEC tissue and known to contribute to osteoclast activation (reviewed in 211). As a result, studies could correlate TNF-a, IL-1α as well as IL-6 directly to the severity of bone destruction in MEC patients [80, 212–214]. In accordance with this, an in vivo model of MEC linked the presence of proinflammatory macrophages and T-cells to bone resorption of MEC [22, 23]. This cytokine driven maturation might explain the observed correlation between bone resorption and LPS [14], without induction of RANKL by LPS [17]. The central role of TLR4 was further confirmed by an TLR4 knockdown in an animal model of acquired MEC, which reduced the amount of osteoclast formation and bone destruction significantly [35].

Other cytokines were investigated as well but no clear evidence was found for their correlation to bone resorption in the clinic. For example the macrophage-colony stimulating factor, crucial for complete osteoclastgenesis (reviewed in 215), was found to be overexpressed in MEC specimens compared to normal external meatal skin [193] in Th_1_ cells [193] and IL-1β known to promote osteoclast activation [216] and LPS induced bone resorption in animal models [217], is also upregulated [81]. But no further investigations were undertaken to elucidate the role of M-CSF or IL-1β driven osteolysis in MEC.

Different drugs are available to target these cytokines. The processing of IL-1β can be inhibited by several inflammasome inhibitors (reviewed in 218). Some of them have already been applied clinically i.e. parthenolide to treat various inflammatory diseases [219], pralnacasan [220] or dapansutrile [221] to treat rheumatoid arthritis and tranilast shown to be useful against asthma and keloid scars [222]. For the IL-1 receptor downstream signalling, diacerein used against osteoarthritis in clinic might be a useful approach [223]. Clinical inhibition of IL-6 signalling is achieved by several clinically approved JAK inhibitors from the 1st and especially the more specific 2nd generation, as well as the STAT3 inhibitors (reviewed in 224). The most popular among these drugs are upadacitinib used in patients with inflammatory driven diseases across gastroenterology, dermatology and rheumatology [225], filgotinib [226] or peficitinib [227] efficacious in patients with rheumatoid arthritis and the STAT3 antisense oligonucleotide AZD9150 [228] applied against cancer. As regards TNF-α, numerous small molecules are suspected to be effective [229] but only Iguratimod [202] was developed to the clinical stage testing where it was used to treat rheumatoid arthritis [230]. M-CSF is suspected to play an important role in osteoclastogenesis in MEC, and M-CSF can be decreased by bisphosphonates [199] and the pharmaceutical AZD4547 [200]. M-CSF signalling might be further decreased by the novel M-CSFR inhibitor pexidartinib which has been clinically approved for cancer therapy [231].

Even though it is known that COX-2/PGE2 plays a central role in osteoclast activation in general and in LPS induced osteoclast activation in particular [232] it has not been investigated in MEC driven osteolysis so far. Upregulated expression of the enzyme COX-2 needed for PGE2 synthesis has been localized in MEC epithelium [233]; the proinflammatory prostanoid receptors EP1-EP3 are upregulated while the anti-inflammatory EP4 receptor is downregulated [234] which suggests an enhanced proinflammatory PGE2 signalling in MECs. Numerous well characterized COX-2 inhibitors are available and in particular, the ones specific to COX-2 might offer a treatment opportunity for MEC. Among them are drugs like celecoxib [235], etoricoxib [236], rofecoxib [237] and meloxicam [238] all proven to be useful against different forms of arthritis.

The presence of matrix metalloproteinases (MMPs) might be a driving force behind the enzymatic digestion of bone in MEC disease. The presence of MMPs play an important role in resorption of the bone by decomposing the organic components of bone tissue. In accordance with the well documented induction of MMP-2 and MMP-9 via NF-κB and AP-1, the expression of MMP-2 as well as MMP-9 is generally upregulated in comparison to healthy skin and correlated to the level of inflammation [239–242]. The high expression of MMP-2 was almost exclusively found in fibroblasts or in the inflammatory cells of the perimatrix [243] and positively correlated to bone destruction in patients [212, 239] and the expression of MMP-9 was found to correlate to the level of osteolysis as well [212, 244].

Due to their attractiveness as pharmaceutical targets, numerous inhibitors of MMPs were developed in the past and tested in clinical trials [244–246]. All these drugs were applied on solid tumors to prevent angiogenesis or metastasis e.g. chlorotoxin inhibits MMP-2 [247] or broad spectrum inhibitors acting on MMP-2 and MMP-9 simultaneously like diazepinomicin [248], rebimastat [249], marimastat [250], arctigenin [251]. Unfortunately, many drugs have failed safety trials predominantly due to adverse effects coupled to their systemic use, hence it is logical to assume a topical application may resurrect numerous drugs that have failed to reach the market. Interestingly, in vitro experiments with Vitamin D3 showed a decrease of MMP-2 and MMP-9 in inflamed fibroblasts [252] and in MEC Keratinocytes [253]. Since Vitamin D3 might has been used therapeutically against different forms of arthritis [254], it might offer an additional and much safer therapeutic opportunity.

**Fig. 3:**
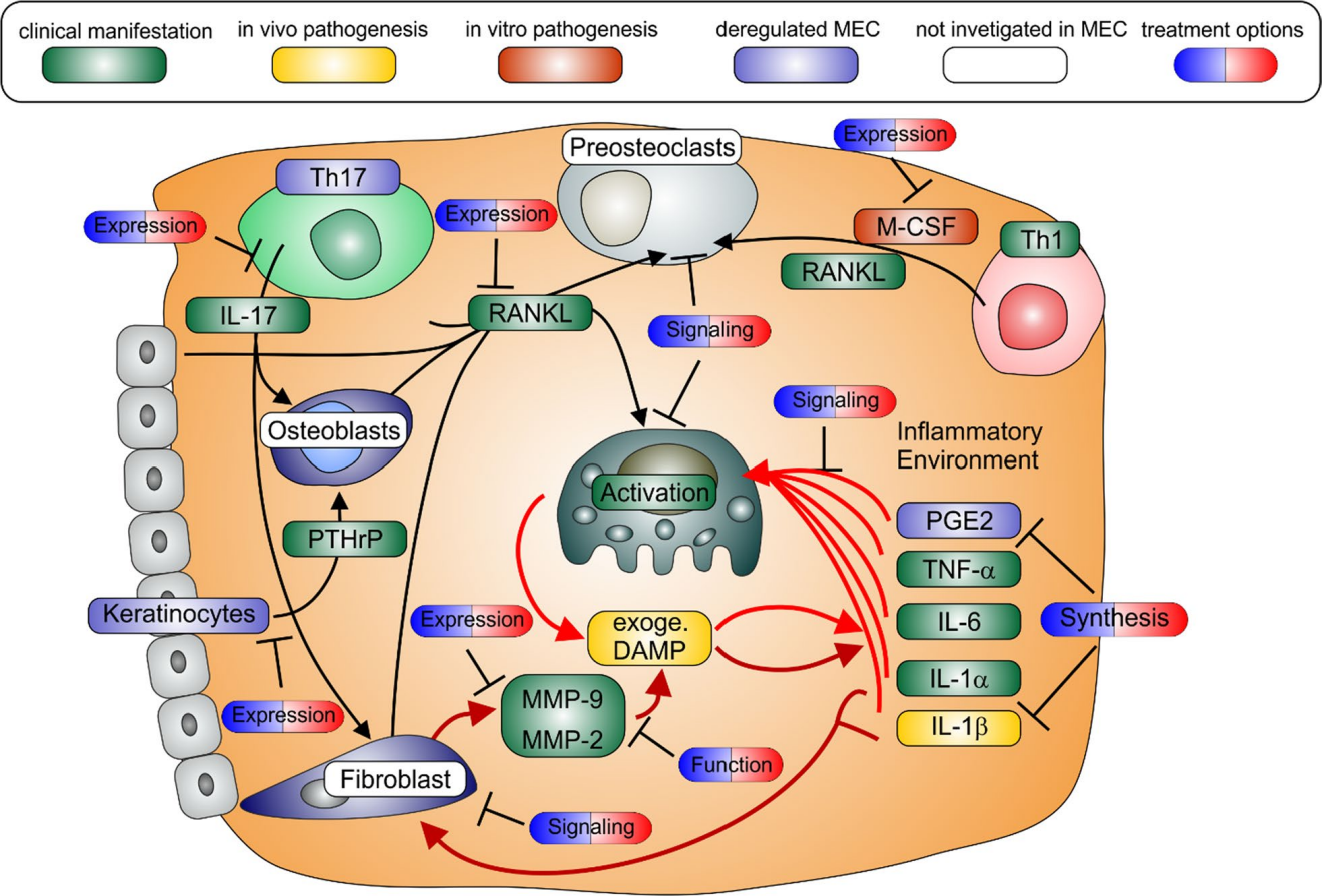
Osteolytic signalling network in MEC. The colour coding is the same as in Fig. 1. The two spirals of doom (red arrows) are interconnected at the generation of exogenous DAMPs derived from ECM tissue destruction

It can be said definitively, that a spiral of doom leading to osteolysis is established in MEC tissue. In highly inflamed MEC tissue the expression of various osteoclastic activators TNF-α /IL-1α/IL-1β/IL-6/RANKL/ PTHrP/M-CSF are induced initially through TREM-1 fuelled TLR4 signalling and subsequently in a paracrine manner. This leads to activation of osteoclasts and subsequent osteolysis. This bone lysis is further amplified by the expression of MMPs in the inflamed MEC niche. As observed for other inflammatory osteolytic diseases (255, reviewed in 256), the DAMPs released from the bone tissue intensifying the TREM-1/TLR4 signalling even more and further boosting the osteoclast activation and MMP secretion (Fig. 3).

### Targeting the fractions of the inflammatory network inducing epidermal proliferation

The most prominent feature of MEC disease is the ongoing proliferation of keratinocytes and the accumulation of keratin debris. Several factors are upregulated in MEC tissue due to its inflammatory state and are known to stimulate the proliferation of MEC keratinocytes e.g. PGE2 [15, 257], TNF-α [258], IL-1α [259] or IL-6 [260]. To reduce the effect of these proliferative factors, PGE2, TNF-α, IL-1α and IL-6 can be targeted by the approaches described in the previous subsection.

The most important stimulation of epidermal proliferation in MEC tissue is induced through paracrine signalling, via a mechanism well known from cutaneous wound healing and skin tissue homeostasis. In this process keratinocytes express PTHrP as well as IL-1α and IL-1β. These proteins will induce the expression of epidermal growth factor (EGF) and keratinocyte growth factor (KGF) in the fibroblasts of the dermis. These factors are vitally important in final induction of epidermal proliferation. Due to the inflammatory processes described above the expression of PTHrP [191], IL-1α [158, 174, 261],] and IL-1β [81, 261] are already upregulated in MEC keratinocytes. The expression of IL-1β might be further augmented by DAMPs activating the inflammasome as demonstrated for MEC keratinocytes [262]. In addition to the upregulation of the IL-1s, the interleukin-1 receptor antagonist (IL-1-RA) showed a decrease compared to healthy skin samples [263], further enhancing the effect. The expression levels of KGF [264, 265] as well as EGF [174] were also enhanced in fibroblasts residing in MEC tissue compared to fibroblasts of healthy skin. The high expression of EGF could be attributed to the fact that fibroblasts derived from healthy skin showed a much weaker expression of epiregulin [266] after in vitro stimulation with IL-1α and/or IL-1β compared to MEC fibroblasts. The increased expression of KGF in patients could be correlated with strong levels of the inflammatory infiltrate [267] and inflammation in general [268]. The paracrine signalling between fibroblasts and keratinocytes is further enforced by the increased expression of the corresponding receptors KGFR [241] and EGFR [269, 270] in the epithelium of MEC. In particular, KGF was found to be one of the main players in MEC development and able to induce MEC formation solely by its overexpression [271–274]. In accordance with that, the expression of KGF and KGFR could be directly correlated to epidermal proliferation of MEC tissue [241, 275, 276] as well as to proliferation of stem/progenitor cells [273, 274] which might also explain the correlation between KGF/KGFR and pathogenesis, as well as clinical recurrence of MEC after surgery [264, 275].

This suggests, that due to keratinocyte hyperproliferation and the high level of inflammation in MEC tissue, the fibroblasts receive excess PTHrP as well as IL-1α and IL-1β. This establishes a spiral of doom with increased paracrine signalling via EGF and KGF which further enhances the amount of PTHrP as well as IL-1α and IL-1β secreting keratinocytes and keratin debris which further increases inflammation by PRR signalling. All this makes this paracrine positive feedback loop spin completely out of control. PTHrP as well as IL-1α and IL-1β can be targeted by the means described in the previous subchapter. For KGF, the main driver of epidermal proliferation, targeted therapies via entrectinib [277] and larotrectinib [278] are available. The upregulation of different cytokines not only directly stimulates epidermal proliferation but more importantly fuels the paracrine feedback loop, which supplies growth factors to the keratinocyte. This will lead to more keratin debris and DAMP respectively, resulting in a self-enhancing inflammation. The significance of the contribution of hyperproliferation to MEC pathogenesis is reflected in the correlation between upregulation of Ki67 and downregulation of p27 relative to meatal skin tissues and the worsening of the prognosis in terms of bone erosion and recurrence rates [279]. In addition to this, paracrine signalling experiments on a complex in vitro model, which utilized a co-culture of fibroblast and keratinocytes derived from MEC, demonstrated an impressive reduction of viability and increase of apoptosis in karatinocytes after application of the COX-2 inhibitor [280].

### Pharmaceutical targets responsible for angiogenesis in MEC

To support the described hyperproliferation, the abnormal growth of new vessels is crucial and accordingly the perimatrix of MEC contained significantly more micro vessels in the subepithelial connective tissue compared to ear canal skin [281, 282]. Since EGFR is upregulated [269, 270] and promotes angiogenesis [283], it is not surprising, that the increased density of micro vessels correlated with the increased expression of EGFR in inflamed MEC [269]. Due to its clinical significance, several small molecule inhibitors of EGFR signalling were developed and approved for clinical application. The most common are afatinib [284], neratinib [285], dacomitinib [286] and gefitinib [287] all approved to be effective against EGFR driven carcinomas.

Other drivers of angiogenesis have been investigated as well. The most prominent is the vascular endothelial growth factor (VEGF), for which expression is upregulated in compared to normal skin [241, 282]. A correlation between NF-κB driven inflammation and VEGF was shown to promote the neovascularisation in MEC tissue [84]. VEGF signalling can be inhibited by approved drugs on the level of intracellular signalling by drugs like sunitinib [288] or axitinib [289], which are approved for treatment of renal cell carcinoma or nintedanib applied against pulmonary fibrosis [290] or lucitanib used in clinical trials against cancer [291].

Indeed other growth factors induced by inflammation and related to angiogenesis like bFGF [292] or TGF-β [293, 294] are also upregulated in MEC epithelium [295, 296], respectively. Since it became clear that stromal expansion plays a rather secondary role in MEC pathogenesis, these factors were neglected in MEC research. In the light of the paracrine positive feedback loop between epidermal and stroma cells, the stromal proliferation and these factors respectively may again be invoked as pharmaceutical targets.

Due to the role of FGF/FGFR in various diseases, numerous pharmaceuticals targeting the signalling pathways have been tested [297]. Even though only ponatinib is approved for the treatment of chronic-phase chronic myeloid leukemia [298], there are several under clinical investigation in phase III trials, i.e. orantinib [299], cediranib [300] or lucitanib [291] used against cancerous disease or nintedanib to treat pulmonary fibrosis [290], interestingly the last two drugs work also as VEGFR inhibitors. For TGF-β inhibition only two clinical drugs are available so far, one is the antisense oligonucleotides AP-12009 which act directly against the mRNA of TGF-β [301] and the TGFB receptor antagonists like galunisertib, which are both used against carcinomas [302].

Other angiogenic factors e.g. IL-8 and COX-2 are highly expressed in the inflamed MEC tissue [38, 233, 303] respectively. An interesting study showed, that id1 increased endothelial proliferation in MEC by upregulating the expression of COX-2, VEGF via ERK1/2 and IL-8 through NF-κB [304]. Hence drugs targeting id1 not only reduce epidermal proliferation but angiogenesis as well.

Other upregulated angiogenic factors upon inflammation are the already mentioned proteinases MMP-2, MMP-9. These MMPs are well known to be crucial for the decomposition of the extracellular matrix making the tissue susceptible for vascular outgrowth. An immunofluorescence correlation study in cholesteatoma tissue demonstrated, that correlations between NF-κB and these MMPs causes the intensification of angiogenesis in MEC [84] and can be targeted by drugs described in the previous subchapter.

**Fig. 4:**
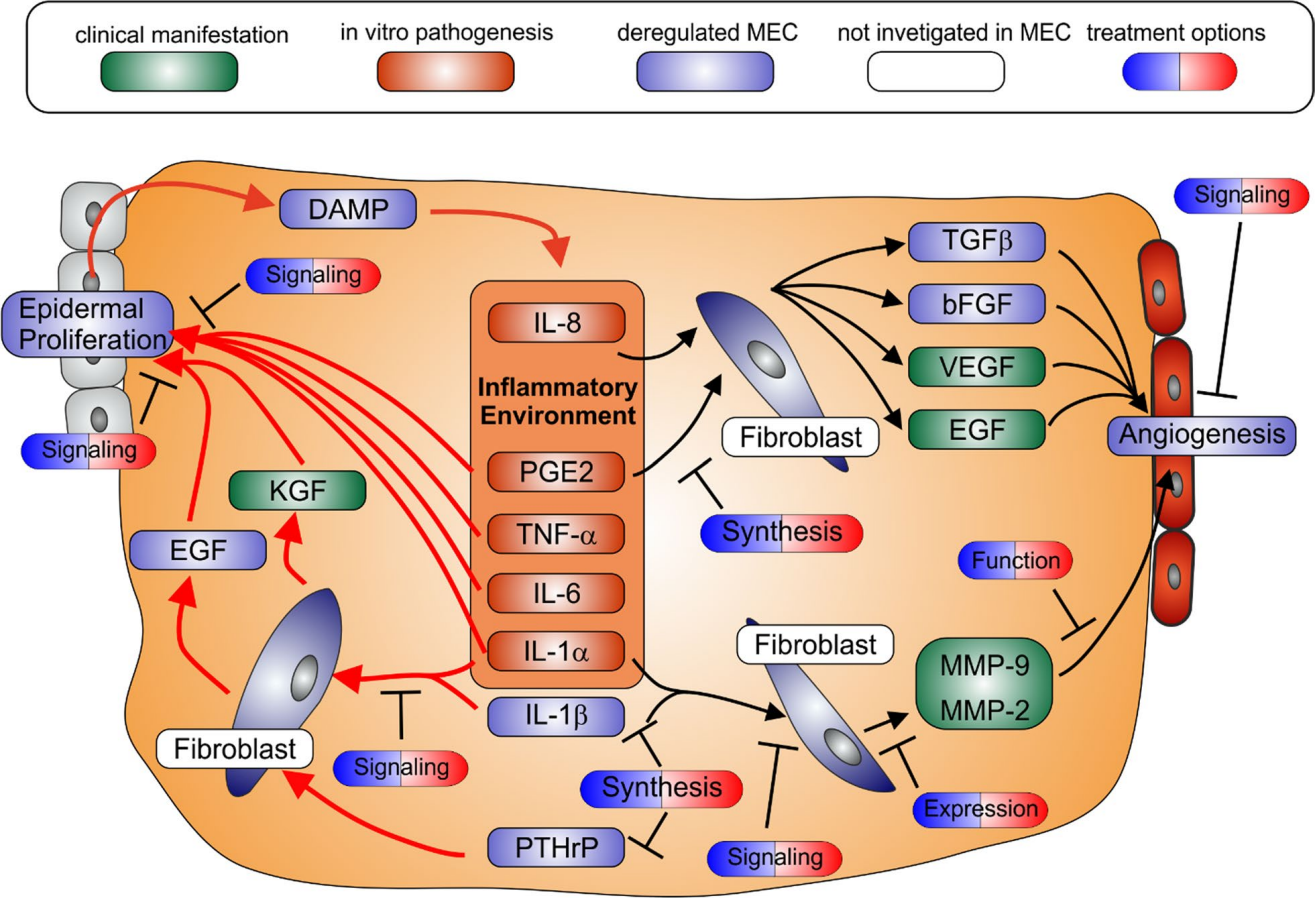
The signalling leading to epidermal proliferation and angiogenesis. The colour coding is the same as in Fig. 1. The spiral of doom involving the increased epidermal proliferation is shown in red and revolves around endogenous DAMP generated from dying epithelial cells

In sum, the increase of blood supply to the MEC is supported by its own inflammation by the upregulation of EGFR, VEGF, MMPs and presumably COX2, bFGF or TGF-β. This suggests, that this increased vascularisation by inflammation is a self-fuelling spiral of doom, since it not only enables the hyperproliferation but also provides better access for the T-cells, mast cells or monocytes/macrophages to the MEC further enhancing the inflammation (Fig. 4).

## Conclusion

To date, numerous studies to inhibit the formation or recurrence of MEC have been undertaken. Unfortunately, many of these have focused on reducing the (already triggered) hyperproliferative behaviour of MEC epithelial cells, via cytotoxic approaches usually applied to cancer e. g. photodynamic therapy [305] or cytostatic 5-fluorouracil [306]. Even though 5-fluorouracil made its way into clinical trials [307, 308] the results were insufficient to establish its application in routine clinical practice for MEC. We suggest long term application of non-cytotoxic substances targeted on the inflammatory pathways described in this review might well be a superior strategy. In accordance with long term application, gradually absorbed formulations comprising non ototoxic carrier substances like hydrogels based on chitosan glycerophosphate and hyaluronic acid [309], are mandatory, but are not within the remit of this review. As regards topical application, the MEC site shows characteristics of an open wound rather than an intact epithelial barrier. Hence, topical application might work for many drugs normally not able to cross the intact epithelial barrier. Indeed, local topical application, would revive the prospect of many drugs described in this chapter, which showed severe side effects in clinical trials due to systemic application.

In this review we have proposed numerous molecular targets which appear to be vital to the deregulated inflammation in MEC, and known to be correlated to MEC pathogenesis. Unfortunately, the connection between certain targets e.g. AP-1 were not investigated in such detail, hence specific molecular targeted therapy (MTT) approach for them were not included. Other targets like TREM-2, GMCSF, etc. (Additional file 1: Table S1) were clearly correlated to pathogenesis, but for these no drug within the restrictions applied in this review were available up to now. Also the role of cytokines important for Th1 differentiation like IL-12, IL-18 or INF-γ with the last one being also crucial for M1 polarization, have not been investigated in MEC pathogenesis at all, hence we have not suggested MTT for this targets.

**Fig. 5:**
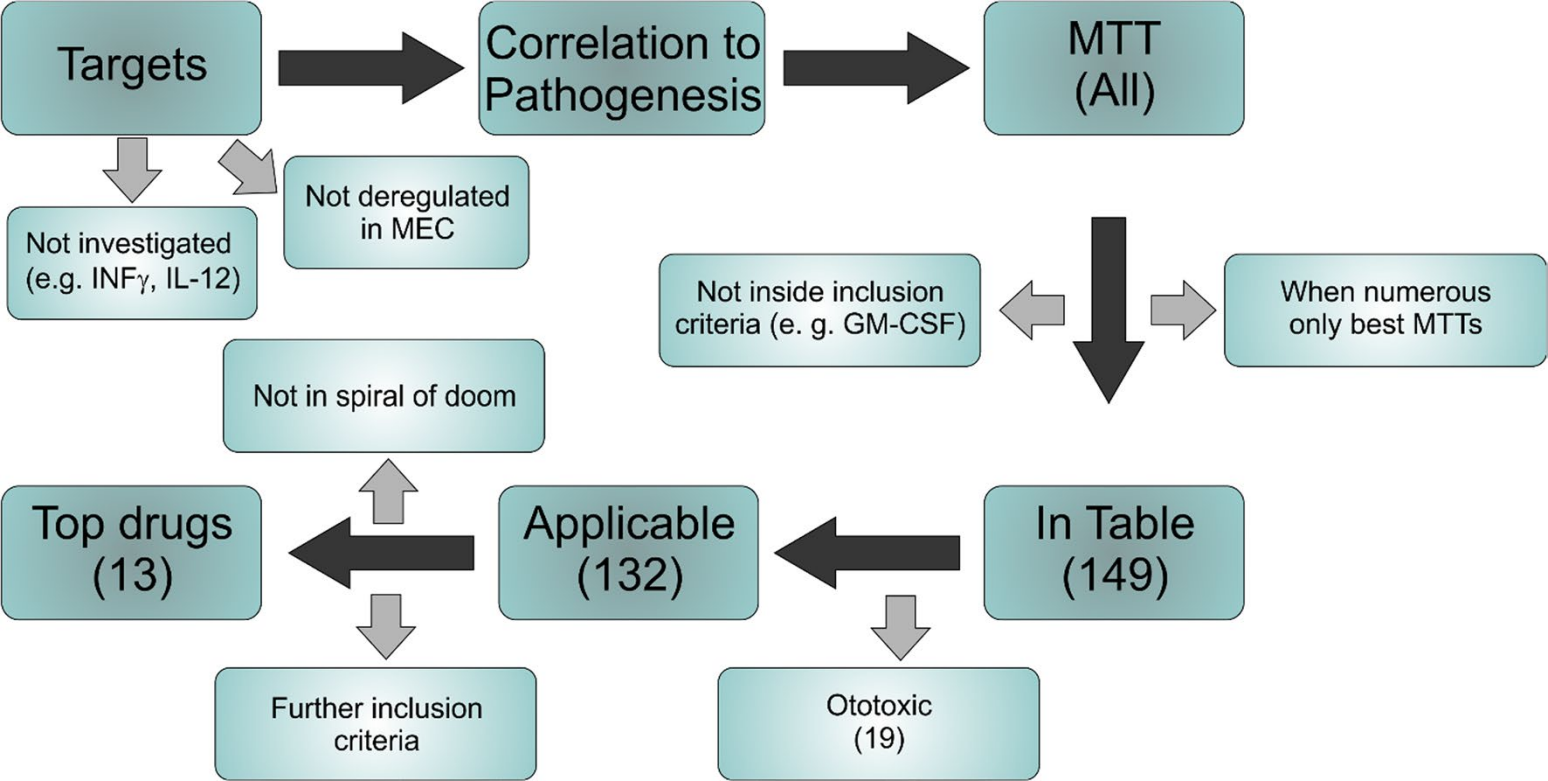
Flow chart of the method used to narrow down possible drugs applicable in a MTT approach for precision medicine for MEC

In the end, we are left with 149 clinically applied drugs against asthma, inflammatory bowel diseases, different forms of arthritis, multiple sclerosis, cancer etc. (Additional file 2: Table S2). We have reduced the number of MTTs or drugs respectively down to a shorter list of top drugs (Fig. 5). This step is crucial, since the MTTs should be tested in an animal model of MEC first before applying for ethical approval for clinical studies, and screening of over a hundred drugs on thousands of animals is neither practical (with the realistic but complex MEC animal models), nor ethically sound.

The mandatory inclusion criteria for a drug is, that the corresponding pharmaceutical approach needs to be part of one of the described self-amplifying spirals of doom (Fig. 5). This is a vital point, since these self-amplifying mechanisms leading to the exaggerated inflammation is fundamental in MEC pathogenesis, epidermal proliferation and osteolysis.

From that group of drugs, all drugs approved for topical application or targeting at least two levels of a vicious circle simultaneously are included. Since the set of top drugs should include a drug for every approach shown in Table 1 we selected the drug not known to induce dermatological side effects, making them unsuitable for topic application to the middle ear or enhancing the likeliness of infection, something undesirable to have in the MEC diseased middle ear. NF-κB has been repeatedly reported to contribute to the initial establishment as well as the fuelling and escalation of the inflammation and is a vital part of the three spirals of doom described, targeting this pathway should be most definitively considered. When careful in vivo evaluation unveiled a couple of MMT able to successfully target MEC, the complex interwoven inflammatory networks described in this review may offer synergistic interaction between these drugs. Hence we suggest investigating this by performing animal experiments enabling calculations of isobolograms. We also want to mention that the MTTs described in Additional file 2: Table S2 are limited due to our inclusion criteria.

**Table 1:** The 13 top drugs most recommended to be investigated in animal models of MEC

Target	Approach	Drug	First approval/stage of trial	Application	Drug type	Ototoxic
TLR4	TLR4 expression	Ibudilast	Phase III	Oral	Small molecule	No
	MD-2-TLR4 antagonist	Taxanes	1995	Oral	Small molecule	No
		Ibudilast	Phase III	Oral	Small molecule	No
	TLR4-signalling	Naloxone	1971	Oral/topic	Small molecule	No
RAGE	RAGE antagonist	Azeliragon	Phase III	Oral	Small molecule	No
TLR4, RAGE, IL-1R, TNFR	BTK inhibitor	Fenebrutinib	Phase III	Oral	Small molecule	No
TLR4 and IL-1R	IRAK inhibitor	PF-06650833	Phase II	Oral	Small molecule	No
TLR4 and TNFR	c-IAP inhibitor	Birinapant	Phase II	Oral	Small molecule	No
TLR4, RAGE, TNFR	Pi3K inhibitor	Duvelisib	2018	Oral	Small molecule	No
Osteolysis/epidermal	Inflammasome	Tranilast	1982	Oral/topic	Small molecule	No
proliferation	Inhibitor	Dapansutrile	Phase II	Oral/topic	Small molecule	No
	IL-1R signalling	Diacerein	2008	Oral/topic	Small molecule	No
	IL-6R signalling	Upadacitinib	2019	Oral	Small molecule	No
	TNF-α signaling	IGURATIMOD	2012	Oral	Small molecule	No

The top drugs were chosen in regard to a fast translation into clinic in accordance with the criteria described in the manuscript

## Supplementary Information

**Additional file 1: Supplementary table 1.** Potential MEC targets which need further investigation regarding new drugs or their role.

**Additional file 2: Supplementary table 2.** All suggested drugs applicable in the middle ear context (light green: double targeting drugs / dark green: drugs double targeting on the same target / * = dizzienes or vertigo).

## Data Availability

All data generated or analysed during this study are included in this published article [and its supplementary information files].

## Abbreviations

MEC: Middle ear cholesteatoma
MTT: Molecular targeted therapies
PAMP: Pathogen associated molecular pattern
DAMP: Damage associated molecular pattern
ECM: Extracellular matrix
LPS: Lipopolysaccharide
Mincle: Macrophage inducible Ca2+-dependent lectin receptor
TLR4: Toll-like receptor 4
RAGE: Receptor for advanced glycation end product
FRET: Förster resonance energy transfer
TREM-1: Triggering receptor expressed on myeloid cells-1
MIF: Macrophage migration inhibitory factor
HIF-1: Hypoxia-inducible factor 1
BTK: Bruton’s Tyrosine Kinase
c-IAP: Cellular inhibitor of apoptosis proteins
HLA-DR: Human Leukocyte Antigen-DR isotype
RANKL: Receptor activator for nuclear factor kappa B ligand
PTHrP: Parathyroid-hormone-related protein
MMP: Matrix metalloproteinases
EGF: Epidermal growth factor
IL-1-RA: Interleukin-1 receptor antagonist
