# Supplementary material for: Review of potential medical treatments for middle ear cholesteatoma
Source: Cell Commun Signal. 2022 Sep 19;20:148. doi: 10.1186/s12964-022-00953-w (PMC9487140; doi:10.1186/s12964-022-00953-w)
Supplement: Supplementary file 2 — Additional file 1: Supplementary table 1. Potential MEC targets which need further investigation regarding new drugs or their role. [file 12964_2022_953_MOESM2_ESM.docx]

Supplementary table 1: potential MEC targets which need further investigation regarding new drugs or their role

| **deleted target** | **reason** | **relation to MEC** |
| --- | --- | --- |
| TREM-2 | no drug | upregulated in MEC / amplifying TLR4 signalling /  correlated to bone decomposition and osteoclast activation in vivo and in clinic |
| GM-CSF | no drug | upregulated in MEC and induced the proliferation of keratinocytes |
| CYLD | no drug | downregulated in MEC /  negatively correlated to activation of NF-κB in MEC epithelium |
| β-catenin | contradicting  reports | downregulation (no effect) /  upregulated (promoting MEC epidermal cell proliferation |
| HLA-DR | no drug | HLA-DR upregulated in MEC /  expressed in macrophages /linked to T-cell activation |
| OPG | no drug | upregulation in some MEC negatively correlated to bone resorption |
| miRNA-17 | no drug | miRNA-17 in exosomes of MEC keratinocytes  induce RANKL in fibroblasts in in vivo model |
| psoriasin | no drug | upregulated in MEC /  promote endothelial proliferation |
| PGF | no drug | upregulated in MEC / driver of skin angiogenesis |
