# Supplementary material for: Review of potential medical treatments for middle ear cholesteatoma
Source: Cell Commun Signal. 2022 Sep 19;20:148. doi: 10.1186/s12964-022-00953-w (PMC9487140; doi:10.1186/s12964-022-00953-w)
Supplement: Supplementary file 3 — Additional file 2: Supplementary table 2. All suggested drugs applicable in the middle ear context (light green: double targeting drugs / dark green: drugs double targeting on the same target / * = dizzienes or vertigo). [file 12964_2022_953_MOESM3_ESM.docx]

Supplementary table 2: All suggested drugs applicable in the middle ear context (light green: double targeting drugs / dark green: drugs double targeting on the same target / * = dizzienes or vertigo)

| **Target** | **Approach** | **Drug** | **first approval /  stage of trial** | **Applikation** | **Drug Type** | **ototoxic** |
| --- | --- | --- | --- | --- | --- | --- |
| Infection | antibiogram | specific antibiotic | - | topic | small molecule | no |
| Inflammation | generall | montelukast | 1998 | oral | small molecule | no* |
| TLR4 | TLR4 expression | Ibudilast | Phase III | oral | small molecule | no |
|  |  | RTL1000 | Phase I | IV | petide | no |
|  | MD-2-TLR4 antagonist | eritoran | Phase III | IV | small molecule | no |
|  |  | JKB-122 | Phase III | oral | small molecule | no |
|  |  | VB-201 | Phase II | oral | small molecule | no |
|  |  | curcumin | Phase III | oral | small molecule | no |
|  |  | taxanes | 1995 | oral | small molecule | no |
|  |  | Ibudilast | Phase III | oral | small molecule | no |
|  |  | dalcetrapib | Phase III | oral | small molecule | no |
|  | TLR4-signalling | resatorvid | Phase III | IV | small molecule | no |
|  |  | resveratrol | 2011 | oral | small molecule | no |
|  |  | alogliptin | 2012 | oral | small molecule | no |
|  |  | naltrexone | 1984 | oral | small molecule | no* |
|  |  | naloxone | 1971 | oral/topic | small molecule | no |
| TREM-1 | TREM-1 expression | curcumin | Phase III | oral | small molecule | no |
|  | TREM-1 antagonist | nangibotide | Phase II | IV | petide | no |
| RAGE | RAGE antagonist | azeliragon | Phase III | oral | small molecule | no |
| Mincle | Mincle expression | curcumin | Phase III | oral | small molecule | no |
|  |  | quercetin | 1953 | oral/topic | small molecule | no |
| Signaling of | BTK inhibitor | acalabrutinib | 2017 | oral | small molecule | no |
| TLR4, RAGE, |  | Ibrutinib | 2017 | oral | small molecule | no |
| TREM-1, IL-1R |  | dasatinib | 2006 | oral | small molecule | yes |
| and TNFR |  | zanubrutinib | 2019 | oral | small molecule | no |
|  |  | fenebrutinib | Phase III | oral | small molecule | no |
|  |  | tirabrutinib | 2020 | oral | small molecule | no |
| Sign. of TLR4 and IL-1R | IRAK inhibitor | PF-06650833 | Phase II | oral | small molecule | no |
| Sign. of TLR4 and TNFR | c-IAP inhibitor | birinapant | Phase II | oral | small molecule | no |
| SIgnalling of TLR4, | Pi3K inhibitor | copanlisib | 2017 | IV | small molecule | no |
| RAGE, TNFR and IL-6-R |  | alpelisib | 2019 | oral | small molecule | no |
|  |  | duvelisib | 2018 | oral | small molecule | no |
|  |  | idelalisib | 2014 | oral | small molecule | no |
| NF-κB | IKK inhinbitor | CHS-828 | Phase II | oral | small molecule | no |
|  |  | VGX-1027 | Phase I | oral | small molecule | no |
|  | inhibitors of | disulfiram | 1949 | oral | small molecule | no |
|  | degradation of IkB | ixazomib | 2016 | oral | small molecule | no |
|  |  | carfilzomib | 2012 | IV | small molecule | no |
|  |  | marizomib | Phase III | oral | small molecule | no |
|  |  | oprozomib | Phase II | oral | small molecule | no |
| HIF-1 | enhancing degradation | tanespimycin | 2010 | oral | small molecule | no |
|  |  | panobinostat | 2015 | oral | small molecule | no |
|  |  | vorinostat | 2007 | oral | small molecule | no |
|  |  | geldanamycin | 2008 | oral | small molecule | no |
|  |  | resveratrol | 2011 | oral | small molecule | no |
|  | inhibition expression | EZN-2208 | Phase II | IV | polymeric drug | no |
|  |  | EZN-2968 | Phase II | IV | antisense oligo | no |
|  |  | panzem | Phase II | oral | small molecule | no |
|  | inhibition transcriptional | panzem | Phase II | oral | small molecule | no |
|  | activity | echinomycin | Phase II | IV | petide | no |
|  |  | amphotericin B | 1958 | oral/topic | small molecule | no |
|  | preventing dimerization | acriflavine | 1912 | oral/topic | small molecule | no |
| id1 | inhibition expression | fucoidan | Phase II | oral/topic | small molecule | no |
|  |  | berberine | Phase III | oral/topic | small molecule | no |
| ICAM-1 | inhibition expression | resveratrol | 2011 | oral | small molecule | no |
|  |  | methotrexate | 1988 | oral | small molecule | no |
|  |  | curcumin | Phase III | oral | small molecule | no |
|  |  | alicaforsen | Phase III | topic | antisense oligo | no |
| HLA-DR | HLA-DR expression | iscador |  |  |  |  |
| Monocyte | inhibition of M1 | bilobalide | Phase II | oral | small molecule | no |
| differentiation | polarization | quercetin | Phase II | oral | small molecule | no |
|  |  | berberine | Phase III | oral | small molecule | no |
|  |  | curcumin | Phase III | oral | small molecule | no |
|  |  | arctigenin | Phase I | oral | small molecule | no |
|  |  | rosiglitazone | 1999 | oral | small molecule | no |
|  | tolerogenic | corticosteroids | 1954 | oral/topic | small molecule | no* |
|  | differentiation of DCs | rapamycin | 1999 | oral | small molecule | no |
|  |  | cyclosporine | 1983 | oral | small molecule | no |
| Adaptive immunity | Th 1 differentiation | beta2-agonists | 1940 | oral | small molecule | no* |
|  |  | progesterone | 1978 | oral/topic | small molecule | no |
|  |  | glucocorticoids | 1948 | oral/topic | small molecule | no |
|  |  | flavocoxid | 2004 | oral | small molecule | no |
|  |  | epigallocatechin | Phase II | oral/topic | small molecule | no |
|  |  | (PDE4) inhibitors | 2010 | oral/topic | small molecule | no |
| Innate and adaptive | Th1 cytokine secretion | Vitamin D3 | 1978 | oral/topic | small molecule | no |
| immunity | degranulation of | cromolyn sodium | 1967 | topic | small molecule | no |
|  | mast cells | lodoxamide | 1993 | oral/topic | small molecule | no |
|  |  | nedocromil | 1999 | oral/topic | small molecule | no |
|  |  | ketotifen | 1999 | oral/topic | small molecule | no |
|  |  | olopatadine | 2004 | oral/topic | small molecule | no |
|  | H1R antagonist | ketotifen | 1999 | oral/topic | small molecule | no |
|  |  | olopatadine | 2004 | oral/topic | small molecule | no |
|  | H2R antagonist | nizatidine | 2004 | oral | small molecule | no |
|  | H4R antagonist | toreforant | Phase II | oral | small molecule | no |
|  |  | JNJ39758979 | Phase II | oral | small molecule | no |
|  |  | ZPL-3893787 | Phase II | oral | small molecule | no |
|  |  | UR-63325 | Phase II | topical | small molecule | no |
| Osteolysis | RANKL signalling | bisphosphonates | 1995 | oral | small molecule | no |
|  |  | AZD4547 | Phase III | oral | small molecule | no |
|  |  | isoflavone | 1999 | oral/topic | small molecule | no |
|  |  | iguratimod | 2011 | oral/topic | small molecule | no |
|  | RANKL expression | bisphosphonates | 1995 | oral | small molecule | no |
|  |  | cabozantinib | 2017 | oral | small molecule | no |
|  | M-CSF expression | bisphosphonates | 1995 | oral | small molecule | no |
|  | M-CSFR inhibitor | pexidartinib | 2019 | oral | small molecule | no |
| Osteolysis/Epidermal | PTHrP expression | cabozantinib | 2016 | oral | small molecule | no |
| prolifaration | Inflammasome Inhibitor | parthenolide | 1994 | oral | small molecule | no |
|  |  | pralnacasan | Phase II | oral | small molecule | no |
|  |  | tranilast | 1982 | oral/topic | small molecule | no |
|  |  | dapansutrile | Phase II | oral/topic | small molecule | no |
|  | IL-1R signalling | diacerein | 2008 | oral/topic | small molecule | no |
|  | IL-6R signalling | upadacitinib | 2019 | oral | small molecule | no |
|  |  | filgotinib | 2020 | oral | small molecule | no |
|  |  | peficitinib | 2019 | oral | small molecule | no |
|  |  | AZD9150 | Phase II | IV | antisense oligo | no |
|  | TNF-α signaling | Iguratimod | 2012 | oral | small molecule | no |
| Osteolysis/ | COX-2 inhibition | celecoxib | 1998 | oral | small molecule | no* |
| Epidermal prolifaration/ |  | etoricoxib | 2004 | oral | small molecule | no |
| Angiogenesis |  | rofecoxib | 1999 | oral | small molecule | no* |
|  |  | meloxicam | 2000 | oral/topic | small molecule | no |
| Osteolysis/Angiogenesis | Exp. MMP-2 and MMP-9 | Vitamin D3 | 1978 | oral/topic | small molecule | no |
|  | MMP-2 inhibition | chlorotoxin | Phase III | IV | petide | no |
|  | MMP-2 and MMP-9 | diazepinomicin | Phase II | oral | small molecule | no |
|  | inhibition | rebimastat | Phase III | oral | small molecule | no |
|  |  | marimastat | Phase III | oral | small molecule | no |
|  |  | arctigenin | Phase I | oral | small molecule | no |
| Epidermal prolifaration | KGFR signaling | entrectinib | 2019 | oral | small molecule | no |
|  |  | larotrectinib | 2018 | oral | small molecule | no |
|  | EGFR signalling | TAS-102 | Phase III | oral | small molecule | no |
|  |  | TQB3804 | Phase I | oral | small molecule | no |
|  |  | erlotinib | Phase I | oral | small molecule | no |
|  |  | gefitinib | 2015 | oral | small molecule | no |
|  |  | afatinib | 2007 | oral | small molecule | no |
|  |  | neratinib | 2020 | oral | small molecule | no |
|  |  | dacomitinib | 2018 | oral | small molecule | no |
| Angiogenesis | VEGFR signalling | sunitinib | 2006 | oral | small molecule | no |
|  |  | axitinib | 2012 | oral | small molecule | no |
|  |  | nintedanib | 2014 | oral | small molecule | no |
|  |  | lucitanib | Phase III | oral | small molecule | no |
|  | FGFR signalling | ponatinib | 2012 | oral | small molecule | no |
|  |  | nintedanib | 2014 | oral | small molecule | no |
|  |  | orantinib | Phase III | oral | small molecule | no |
|  |  | cediranib | Phase III | oral | small molecule | no |
|  |  | lucitanib | Phase III | oral | small molecule | no |
|  | TGF-β expression | AP-12009 | Phase III | IV | antisense oligo | no |
|  | TGF-β-R antagonists | galunisertib | Phase III | oral | small molecule | no |
| Angiogenesis | VEGFR signalling | sunitinib | 2006 | oral | small molecule | no |
|  |  | axitinib | 2012 | oral | small molecule | no |
|  |  | nintedanib | 2014 | oral | small molecule | no |
|  |  | lucitanib | Phase III | oral | small molecule | no |
|  | FGFR signalling | ponatinib | 2012 | oral | small molecule | no |
|  |  | nintedanib | 2014 | oral | small molecule | no |
|  |  | orantinib | Phase III | oral | small molecule | no |
|  |  | cediranib | Phase III | oral | small molecule | no |
|  |  | lucitanib | Phase III | oral | small molecule | no |
|  | TGF-β expression | AP-12009 | Phase III | IV | antisense oligonucleotides | no |
|  | TGF-β-R antagonists | galunisertib | Phase III | oral | small molecule | no |
